# Safely increasing nephro-ureteric stent exchange intervals, resulting in significant cost savings for the interventional radiology suite, a 2-year experience in a tertiary referral centre

**DOI:** 10.1007/s11845-021-02657-5

**Published:** 2021-06-05

**Authors:** Emma Tong, Kate Hunter, Joe Deegan, William C. Torreggiani

**Affiliations:** 1 Department of Radiology, Tallaght University Hospital, Dublin, Ireland; 2 Department of Medicine, Trinity College Dublin, Dublin, Ireland

**Keywords:** Exchange, Nephro ureteric stent

## Abstract

**Aim:**

To evaluate the nephro-ureteric stent (NUS) insertion and exchange practice in a tertiary referral cancer centre, and determine the safety and compliance with current guidelines. We also reviewed if increasing exchange time interval from 6 to 12 weeks was safe, and if this could be adopted into our local guidelines.

**Methods:**

A retrospective review was performed covering 24 months from January 2017 to December 2018. All NUS insertions and exchanges performed in that period were analysed, including the number of exchanges the patient underwent, the time between subsequent exchanges, and the screening time. We also reviewed the indications for stent insertion, possible causes for failed stent exchange, and factors which led to significant delays in stent exchanges for some patients. A scatterplot of screening time versus time in situ was derived and correlation analysis performed using the Pearson coefficient.

**Results:**

Thirty-two patients underwent de novo NUS insertion during the period, and 102 NUS exchanges were performed. The interval between stent exchanges ranged from 1 to 40 weeks, with a mean of 12.3 weeks (SD = 8.96 weeks). Screening time ranged from 33 s to 17 min, with a mean of 3 min 50 s (SD = 3 min 35 s). There were 100 successful exchanges, and two failed exchanges, accounting for 1.9% of total exchanges. In both failed cases, the reason for failed exchange was due to a prolonged period between exchanges (6 months in both cases). The reason for delay for stent exchange was due to non-attendance for scheduled appointments. There was a weakly positive correlation coefficient of 0.06 (screening time versus time period between insertions); however, this was not statistically significant (p = 0.81).

**Conclusion:**

In this retrospective review, we have demonstrated that the recommended 6-week period between stent exchanges is unnecessary in the vast majority of cases, and that a longer interval between NUS exchanges, e.g. 8–12 weeks, is safe for the patient, and reduces screening time. This reduction in procedures also provides a significant potential saving to the radiology department in both monetary expense and limited angiography suite time.

## Introduction

**Fig. 1:**
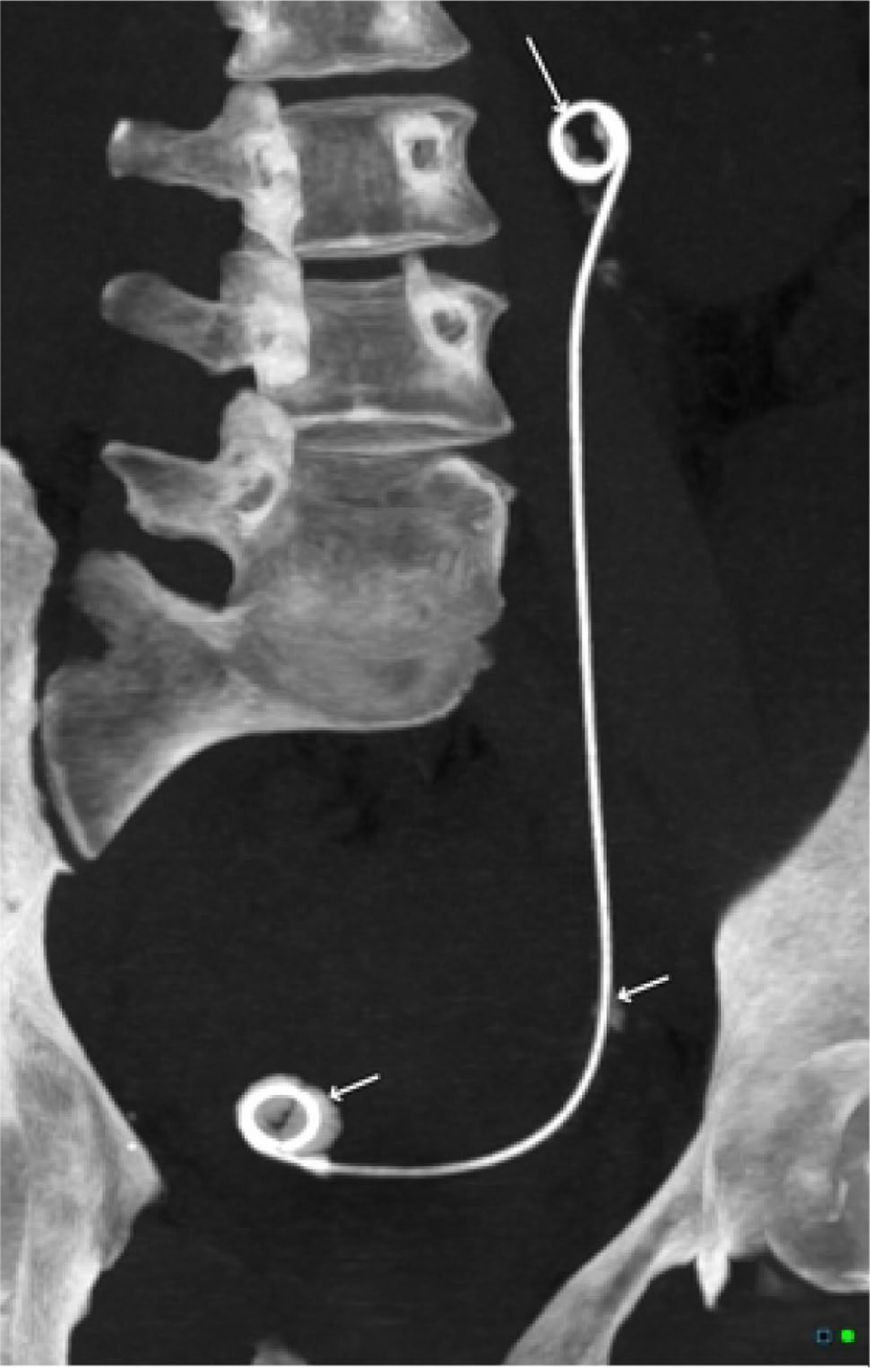
A coronal CT image of a ureteric stent in situ showing encrustations. Case courtesy of Dr Chris O’Donnell, Radiopaedia.org

**Fig. 2:**
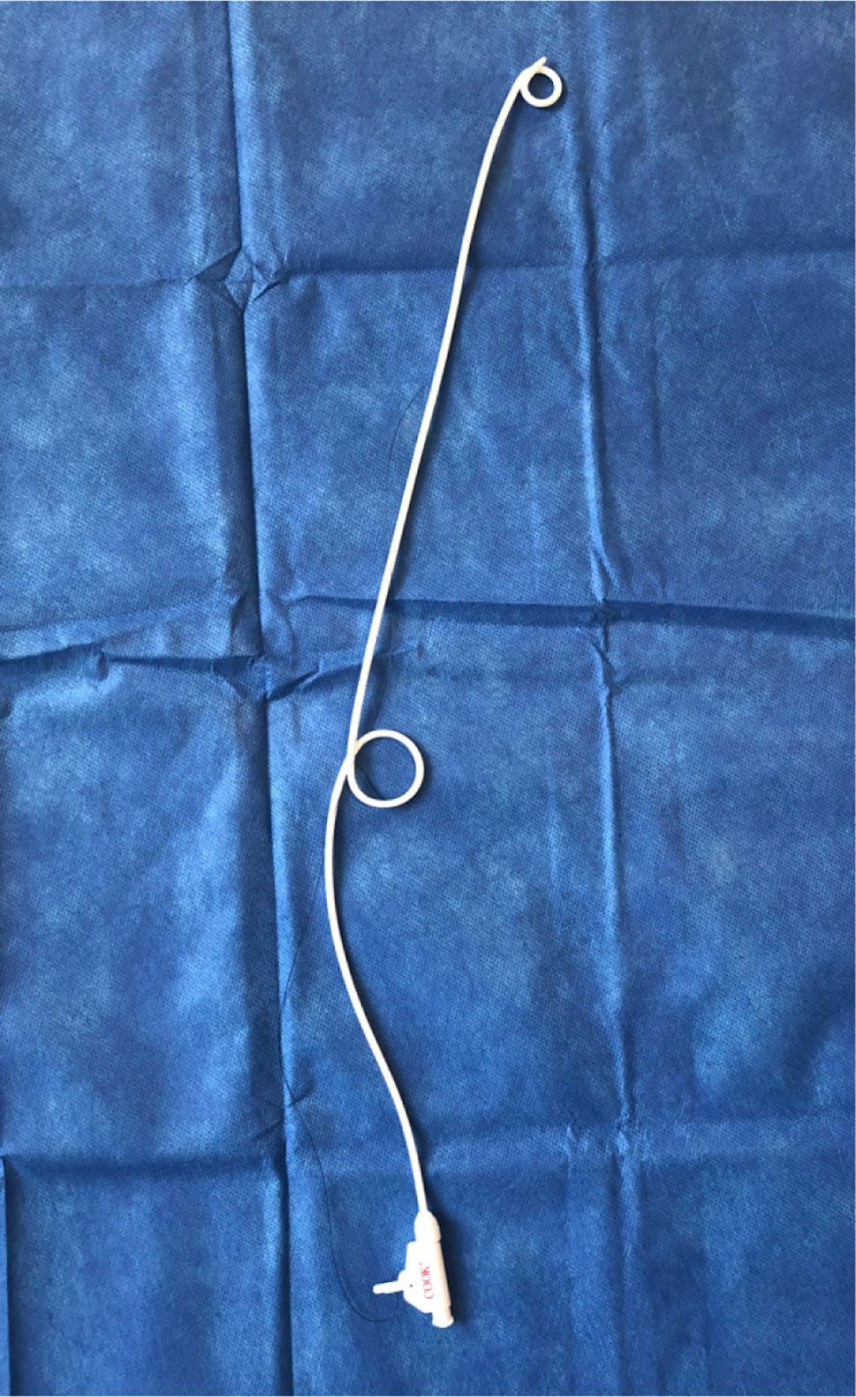
Photo of nephron-ureteric stent used in this study

A nephro-ureteric stent (NUS) allows drainage of urine from the kidney to the bladder, or to an external drainage bag, with curled portions to prevent proximal or distal migration [1]. NUS are used to maintain or re-establish ureter patency by allowing urine to flow through and/or around the stent [2]. While NUS are designed to reduce encrustation, they require regular exchanges in order to prevent this, and also to reduce the risk of infection. Prolonged indwelling time significantly increases the risk of encrustation forming which can ultimately lead to blockage and adverse complications [1]. NUS are indicated in many clinical scenarios, including but not limited to ureteral obstruction (due to nephrolithiasis, compressive mass such as from malignancy, a malignant stricture, or retroperitoneal fibrosis), after surgical ureteral anastomosis (to maintain flow during inflammation and oedema), or as prophylaxis (e.g. before extracorporeal shock wave lithotripsy) [3]. Recent studies have found limited value of NUS placement in ESWL however [4]. NUS placement and exchange are relatively safe procedures, with the majority of complications being minor. The main complications are microscopic haematuria (and occasionally gross haematuria), urinary tract infection (prophylactic antibiotics are sometimes used), stent migration, stent encrustation, and stent retention [5]. Extremely rare complications include stent migration out of the urinary tract, reflux anuria, and arterial-ureteral fistula formation [6, 7] (Figs. 1 and 2).

In cases of stent retention, the typical reason is a significant delay in follow-up NUS removal or exchange, which leads to problematic encrustation. NUS encrustation is caused by uric acid or calcium oxalate precipitation onto the stent surface. While calcium phosphate and ammonium magnesium phosphate (struvite) can also precipitate, this requires a higher pH and therefore usually occurs in association with urinary tract infections involving urea-splitting bacteria which produce ammonia [8]. Severe encrustation in combination with the formation of stones can obstruct the urinary tract and significantly impair kidney function, which can eventually lead to kidney function damage if left untreated. While the most significant risk factor for encrustation is the indwelling time of the stent, there are other confounding factors, including the stent component material, bacterial colonisation, and prior history of urolithiasis [8]. Whilst there are no published protocols on timing of NUS exchange, many authors have published their own guidelines advising that NUS should be exchanged every 6–8 weeks in order to present such complications [9]. The aim of this study was to evaluate NUS insertion and exchange practice in a tertiary referral cancer centre, and determine the safety and compliance with current guidelines (Table 1).

**Table 1:** Indications for NUS Insertion

Indication	Number	%
Bladder cancer	9	27.27
Cholangiocarcinoma	1	3.03
Colorectal cancer	2	6.06
Metastatic cancer with unknown primary	1	3.03
Non-Hodgkin’s lymphoma	1	3.03
Prostate cancer	7	21.21
Rectal cancer	2	6.06
Retroperitoneal fibrosis	1	3.03
Small bowel cancer	1	3.03
Ureteric cancer	1	3.03
Ureteric stricture	1	3.03
Ureteric stricture, 2nd to stone	1	3.03
Ureteric stricture, unknown cause of same	1	3.03
Ureteric TCC (transitional cell carcinoma)	1	3.03
Uterine fibroid	1	3.03
Uterine sarcoma	1	3.03

## Methods

A retrospective review was performed of all NUS insertions and exchanges over 24 months from January 2017 to December 2018 in our interventional radiology department which is based in a tertiary referral centre. All NUS insertions and exchanges performed in that time period were identified and evaluated. Patients were identified for inclusion by manually reviewing the interventional radiology suite logbook and the procedure details. This was cross referenced with manual searches of our Picture Archiving Communication System (PACS). Patient medical charts were also reviewed in cases of missing medical details. The patient name, date of birth, gender, and medical record number were recorded. The database was then anonymised. Review was undertaken of the number of exchanges performed on each patient, the time frame between subsequent exchanges, the screening time, and the indication for NUS placement. Any procedural complications and failed procedures were also evaluated using medical notes, and the official PACS procedure report. All procedures took place in a dedicated interventional suite under aseptic technique, and were performed by, or supervised by, an experienced interventional radiologist. All placements were performed by antegrade access, under fluoroscopic guidance, and under conscious sedation. A scatterplot of screening time versus time in situ was derived and correlation analysis performed using the Pearson coefficient. Statistical analysis was performed with and without outliers (time in situ > 30 weeks). Ethics approval was waived by our research committee as they deemed this study to be an audit (Table 2).

**Table 2:** Average interval and screening time

Indication	Average interval (weeks)	Average screening time
Bladder cancer	11.85	00:02:59
Rectal cancer	15.60	00:03:32
Metastatic cancer with unknown primary	8.50	00:04:30
Ureteric TCC	29.00	00:03:49
Ureteric stricture, unknown cause of same	17.00	00:02:51
Cholangiocarcinoma	12.30	00:02:55
Prostate cancer	15.14	00:06:42
Retroperitoneal fibrosis	13.67	00:02:08
Small bowel cancer	10.00	00:04:26
Colorectal cancer	9.00	00:01:51
Non-Hodgkin’s lymphoma	17.50	00:02:12
Uterine fibroid	23.00	00:03:42

## Results

A total of 32 patients underwent NUS insertion, and 102 NUS exchanges were performed on these 32 patients over the study period. The mean patient age was 68.42 with a range of 28.86 to 89.19 years (SD = 14.35 years). There were 21 male and 11 female patients. The mean number of stent exchanges per patient was 3.22, with a range of 0–12. Four consultant radiologists were involved in this time period. The interval between stent exchanges ranged from 1 to 40 weeks, with a mean of 12.3 weeks, (SD = 8.9 weeks). Screening time ranged from 33 s to 17 min, with a mean of 3 min 50 s (SD = 3 min 35.6 s). There were two failed procedures; the time frame between the previous stents was 6 months in both incidences. Other than the two failed procedures, of which both underwent successful exchange on subsequent visits, there were no significant complications experienced (Table 3).

**Table 3:** Details of failed procedures

Indication	Interval	Screening time	Reason for failure
Bladder TCC	24 weeks	08:06	Severely encrusted stent
Rectal cancer	27 weeks	15:49	Severely encrusted stent

The most common indication for stent insertion was bladder cancer at 27.3% (N = 9), closely followed by prostate cancer at 21.2% (N = 7). The majority of these patients underwent failed JJ stent insertion.

**Fig. 3:**
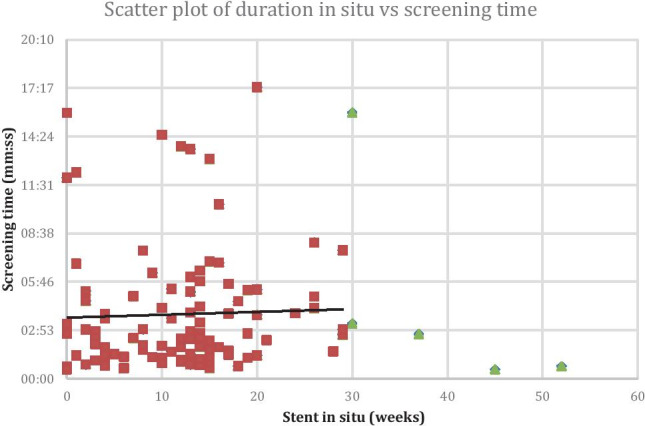
Scatter plot, with stent in situ (in weeks) on the X axis, versus associated screening time (minutes) on the Y axis, with associated trend line. Green data points highlight outliers which were excluded for statistical analysis

There was a weakly positive correlation coefficient of 0.06 of screening time versus time period between insertions; however, the results were not statistically significant with a p value of 0.81. Results were similar for analyses with and without outliers. This suggests that lengthening the time frame between stent exchanges does not have a statistically significant increase in the associated screening time (Fig. 3).

## Discussion

Nephro-ureteric stents were first described by Goodwin et al. and have been in use since 1995 [11]. They are most commonly used in patients with malignancy, as in our patient cohort. NUS are important in relieving urinary obstruction which, if left untreated, can lead to significant renal impairment and ultimately renal failure [10, 11]. These stents require regular and frequent stent exchange to prevent encrustation, blockage, and/or infection, which can cause a significant burden on both the interventional radiology department and on the patient.

Although NUS have been in use for some time, recent developments in stent design, material, and coatings are increasing stent efficacy and safety [12, 13]. Stent manufacturers have moved away from polyethylene and polyurethane to silicone, which is inert and flexible intending to reduce discomfort as well as urothelial erosion [14].

Most of our patients underwent initial failed JJ stent insertion, and the majority of the causes were due to bladder or prostate cancer. Thus, these patients then were referred to interventional radiology for a NUS insertion to manage their symptoms.

Inter-variability between consultants is a limiting factor to our study; however, all of the consultants in our department have many years’ experience in urological interventional radiology and are highly fellowship-trained.

The most recent NHS (National Health Service) tariff payment system, which list a set of prices for most hospital procedures, published in November 2020, estimates that a unilateral percutaneous insertion of a ureteric stent, or nephrostomy, costs up to £1123, and £1545 for a bilateral procedure. Based on a 6 weekly exchange, this could cost up to £10,000–14,000 approximately over 12 months, providing there are no complications. This cost could be halved if the exchange rate was doubled to 12 weeks [15].

## Conclusion

In this retrospective review, we have found that a 12-week exchange time is both safe and effective in our patient cohort. Patients who had longer time frames between exchanges did not experience statistically significant longer screening times. In fact, our longest time frame was 44.4 weeks with an associated screening time of 33 s. We propose that the recommended 6-week period between exchanges is unnecessary in the vast majority of cases of patients with no history of complicated stent exchanges, and that an increased interval between NUS exchanges is safe and reduces the number of procedures for the patients and the IR department. This may also reduce the radiation dose to the patient. A longer time frame between exchanges may also provide a potential saving to the radiology department in both equipment expense and angiography suite time. We have adopted this into our routine policy, and after an uncomplicated stent exchange, the next routine exchange is scheduled for 12 week’s time.
